# Tennessee Dental Association—Resolutions of Respect to Dr. W. H. Morgan

**Published:** 1902-01

**Authors:** 


### TENNESSEE DENTAL ASSOCIATION—RESOLUTIONS OF RESPECT TO DR. W. H. MORGAN.

WHEREAS, With profound regret the Tennessee Dental Association is called upon to notice the death of Dr. W. H. Morgan, an old, tried, and faithful member, and it is, therefore, meet and fitting that it should place on record its appreciation of his long and faithful services as a member, and of his far-reaching, earnest, and valued services to the profession he loved.

His great earnestness in professional work, his faithfulness as a member and officer of this Association, his manliness and friendliness, well merit our most profound appreciation and respect, and his title to the distinction of being honored as the "Father of dentistry in Tennessee."

*Resolved,* That by the death of Dr. Morgan the dental profession at large has lost a great man, and this society a fast and good friend.

*Resolved,* That, bowing in submission to Him who doeth all things well, we hereby express our heartfelt sympathy to his bereaved family; and be it further

*Resolved,* That a copy of these resolutions be transmitted to his family and published in the dental journals.

J. L. MEWBORN,
J. P. GRAY,
A. R. MCCURDY,
*Committee.*

